# The role of anxious distress in immune dysregulation in patients with major depressive disorder

**DOI:** 10.1038/s41398-017-0016-3

**Published:** 2017-12-08

**Authors:** Roxanne Gaspersz, Femke Lamers, Gayle Wittenberg, Aartjan T. F. Beekman, Albert M. van Hemert, Robert A. Schoevers, Brenda W. J. H. Penninx

**Affiliations:** 1 Department of Psychiatry, Amsterdam Public Health Research Institute, VU University Medical Center, Amsterdam, The Netherlands; 2 Janssen Research & Development, LLC, Titusville, NJ USA; 3 Department of Psychiatry, Leiden University Medical Center, Leiden, The Netherlands; 4 Department of Psychiatry, University of Groningen, University Medical Center Groningen, Groningen, The Netherlands

## Abstract

Although depression with anxious distress appears to be a clinically relevant subtype of major depressive disorder (MDD), whether it involves specific pathophysiology remains unclear. Inflammation has been implicated, but not comprehensively studied. We examined within a large MDD sample whether anxious distress and related anxiety features are associated with differential basal inflammation and innate cytokine production capacity. Data are from 1078 MDD patients from the Netherlands Study of Depression and Anxiety. In addition to the DSM-5 anxious distress specifier, we studied various dimensional anxiety scales (e.g. Inventory of Depressive Symptomatology anxiety arousal subscale [IDS-AA], Beck Anxiety Inventory [BAI], Mood and Anxiety Symptoms Questionnaire Anxious Arousal scale [MASQ-AA]). The specifier was constructed using five self-report items from the IDS and BAI. Basal inflammatory markers included C-reactive protein (CRP), interleukin (IL)-6 and tumor necrosis factor (TNF)-α. Innate production capacity was assessed by 13 lipopolysaccharide (LPS)-stimulated inflammatory markers. Basal and LPS-stimulated inflammation index scores were created. Basal inflammation was not associated with anxious distress (prevalence = 54.3%) in MDD patients, except for a modest positive association for BAI score. However, anxious distress was associated with higher LPS-stimulated levels (interferon-γ, IL-6, monocyte chemotactic protein (MCP)-1, macrophage inflammatory protein (MIP)-1α, matrix metalloproteinase (MMP)-2, TNF-α, LPS-stimulated index). Other anxiety indicators (anxious distress specifier score, BAI, MASQ-AA) were also associated with increased innate production capacity. Within a large MDD sample, the anxious distress specifier was associated with increased innate cytokine production capacity but not with basal inflammation. Results from dimensional anxiety indicators largely confirm these results. These findings provide new insight into the pathophysiology of anxious depression.

## Introduction

Major depressive disorder (MDD) is often accompanied by anxious features that can have a negative effect on the outcome of depression^1^. Patients with MDD plus anxiety symptoms were found to have poorer course trajectories^1,2^, more suicidal ideation^3–6^ and worse treatment outcomes^7–10^ than patients with solely MDD. Therefore, it is relevant to assess the pathophysiology of this anxious subtype of MDD more closely. Studies have shown that anxious depressed populations have distinct neurobiological correlates when compared to non-anxious depressed populations^11^, for example stronger dysfunctions of the hypothalamic–pituitary–adrenal axis^12–14^, increased neural activation during functional magnetic resonance imaging cognitive control tasks due to hypervigilance^15^ and deficits in working memory activation^16^. Lately, inflammation has gained interest as another important biological dysregulation in depression.

Inflammation is one of the mechanisms implicated in the pathophysiology of depression^17–19^, as it is found to be more often present in depression than healthy controls in several meta-analyses^20–22^. Most studies have assessed basal inflammation^20–22^. Innate cytokine production capacity, i.e. responsivity of the immune system, has been much less studied but associated with depression as well^23^. Inflammation is also implicated in the pathophysiology of treatment-resistant depression^19,24–26^ and the pathophysiology of anxiety disorders^23,27–29^. It has been hypothesized that anxious depression could be seen as an inflammatory phenotype since it shares common pathophysiological pathways with inflammatory states^30^, but the causality between depression and anxiety with inflammation remains unclear. Miller and Raison^17^ suggest in their review that the hypervigilance characteristic of anxiety disorders might be part of the ‘pathogen host defence’ hypothesis, where inflammatory systems are activated in response to stressors leading to the development of depression^17^. In addition, inflammasomes—protein complexes that recognize various stressors and subsequently trigger activation of cytokines—may also play an important role in the link between inflammation and anxiety and depression^17,31–33^. Conversely, inflammatory processes in the brain may affect metabolic and molecular pathways that influence neurotransmitter systems (e.g. monoamines and glutamate) which ultimately affect neurocircuits that regulate behavior relevant for anhedonia and anxiety^17,34^. To date, only two studies have examined the link between inflammation and depression with concurrent anxiety. One study showed higher monocyte counts in patients with MDD and moderate-severe to severe anxious distress than in those with mild to moderate anxious distress^35^. The other study found decreased venous blood basophil counts and increased fragmented neutrophils in patients with MDD and high levels of anxiety^36^. The findings of these studies reflect alterations in white blood cell subset counts and indirectly may point to alterations in the immune system, suggesting the role of inflammation in the development of anxious depression.

It is important to evaluate both basal inflammation as well as innate production capacity, as these cover different aspects of the immune system. Basal circulating inflammation levels are usually low, have high within-person variability^23^, and are highly influenced by lifestyle and health factors^20,23^. Innate production capacity can be evaluated by the expression of inflammatory markers in response to ex vivo stimulation of blood by lipopolysaccharide (LPS)^37^. This production capacity may provide more insight into the functioning of the immune system^37^ as it mimics the natural environment more closely^38^ and is known to be under strong genetic control^39^. Our group previously compared cytokine production capacity and basal inflammation between persons with a current or remitted depressive or anxiety disorders and healthy controls^23^. Since anxious distress was not previously examined but appears to be a clinically relevant subtype of MDD, we now examined more specifically whether anxious distress and related anxiety features are associated with differential basal inflammation and innate cytokine production capacity in a large MDD sample. This study thus contributes to new insight into the pathophysiology of anxious depression.

The investigation of differential inflammation in MDD with concurrent anxiety features is complicated because different diagnostic criteria have been used^40^. Levels of concurrent anxiety in MDD are often determined by established cut-off scores on different rating-scales^40^. This leads to inconsistent evidence, but the new DSM-5 anxious distress specifier could establish more uniformity to studies on MDD with concurrent anxiety. Our previous work has shown that the DSM-5 anxious distress specifier is a clinically valid construct, and that it outperformed the comorbidity indicator of DSM-IV-based anxiety disorder diagnoses as a longitudinal predictor of important clinical^2^ and treatment^10^ outcomes. We feel, therefore, that it is important to examine whether the anxious distress specifier is characterized by an underlying biological profile, which could contribute to its prediction of poor clinical and treatment outcomes.

The current study examines within a large cohort of depressed patients whether anxious distress and related anxiety features were associated with differential basal inflammatory markers and innate cytokine production capacity. The DSM-5 anxious distress specifier was constructed by items of standard self-report instruments that approximated the DSM-5 criteria of the anxious distress specifier. Besides the new DSM-5 anxious distress specifier, we examined various dimensional anxiety indicators to check for consistency of findings across different anxiety measures.

## Materials and methods

### Study sample

Data were from the baseline assessment (September 2004 to February 2007) of The Netherlands Study of Depression and Anxiety (NESDA), a longitudinal cohort study examining the course and consequences of depressive and anxiety disorders^41^. The sample consisted of 2981 participants (18–65 years) with a current or prior history of depressive and/or anxiety disorder and healthy controls. To reflect different settings and stages of psychopathology, participants were recruited from the community (19.0%), primary care (54.0%) and specialized mental health-care settings (27.0%). Exclusion criteria were insufficient command of the Dutch language or a primary clinical diagnosis of other severe psychiatric conditions. Data collection included an extensive interview, blood collection, medical assessments and self-reported questionnaires. The NESDA project was approved by the Ethical Committee of all participating universities and all participants provided written informed consent.

For the current study, we included 1115 patients with a current (6-month recency) major depressive disorder (MDD) diagnosis, assessed using the DSM-IV-based Composite International Diagnostic Interview (CIDI, version 2.1)^42^. Of these patients, 25 (2.2%) had incomplete data on the DSM-5 anxious distress specifier, and 12 (1.1%) had no data available on both basal and innate inflammatory markers, leaving 1078 MDD patients eligible for analysis. The primary focus of this study is the anxious distress specifier. However, our previous work showed that overlap between presence of the anxious distress specifier and comorbid DSM-IV-based anxiety disorder diagnoses is poor (Cohen kappa = 0.09)^2,10^, indicating that these concepts are not directly comparable. To provide a comprehensive overview of anxiety constructs in relationship to inflammation markers, we also included other anxiety constructs in addition to anxious distress. Patients with comorbid DSM-IV-based anxiety disorder diagnoses were therefore not excluded from the sample. The included MDD patients did not differ from the remainder of the total NESDA sample in terms of sex and physical activity, but were a little younger (40.9 ± 12.0 vs. 42.4 ± 13.6 years, *P* = 0.003), had slightly less educational years (11.7 ± 3.2 vs. 12.4 ± 3.3 years, *P* < 0.001), were more current smokers (*n* = 482, 44.7% vs. *n* = 667, 35.0%, *P* < 0.001), had a higher alcohol intake (>14/21 [female/male] drinks/week: *n* = 42, 3.9% vs. *n* = 71, 3.7%, *P* < 0.001) and had a somewhat higher BMI (25.9 ± 5.4 vs. 25.4 ± 4.8 kg/m^2^,* P* = 0.011).

### Anxious distress specifier

The *anxious distress specifier* was constructed by five self-reported items from the Inventory of Depressive Symptomatology (IDS)^43^ and the Beck Anxiety Inventory (BAI)^44^ that matched directly with the five criteria for the DSM-5 anxious distress specifier. Both questionnaires assess symptoms in the past week on a 0–3 (not at all-severe) scale. Symptoms were considered present when scored ≥2 (i.e. moderate or severe). According to the DSM-5 criterion, the anxious distress specifier was present when a participant endorsed ≥2 of the following symptoms: (1) feeling keyed up or tense (IDS item 7); (2) feeling unusually restless (IDS item 24); (3) difficulty concentrating because of worry (IDS item 15); (4) fear that something awful might happen (BAI item 5); (5) feeling that the individual might lose control of himself or herself (BAI item 14). In addition to the presence of the specifier (dichotomous indicator), the specifier score was also determined (continuous indicator; range 0–15). Our previous work showed an adequate internal consistency (Cronbach’s *α* = .71)^2^, and the specifier had predictive validity for subsequent course and treatment response in depressed patients^2,10^.

### Other anxiety indicators

*Current (6-month recency) anxiety disorder diagnoses* were assessed using the CIDI^42^ and included Social Phobia, Panic disorder with or without Agoraphobia, Agoraphobia and Generalized Anxiety Disorder. A count of the number of anxiety disorders was calculated (range 0–3).

*Different anxiety (sub)scales* were assessed: The Inventory of Depressive Symptomatology anxiety arousal subscale (IDS-AA)^45^, BAI^44^, Fear Questionnaire (FQ)^46^, Mood and Anxiety Questionnaire Anxious Arousal subscale (MASQ-AA)^47^, Anxiety Sensitivity Index (ASI)^48^ and the Penn State Worry Questionnaire anxiety subscale (PSWQ)^49^. Some of these scales focus more on somatic anxiety symptoms (i.e. IDS-AA, BAI and MASQ-AA), others more on cognitive anxiety symptoms (i.e. anxious distress specifier, FQ, ASI, PSWQ).

### Basal and innate inflammatory markers

*Basal inflammatory markers* included C-reactive protein (CRP), interleukin (IL)-6 and tumor necrosis factor (TNF)-α. Fasting blood samples were obtained by laboratory staff (8–9 am) and kept frozen at −80°C. High-sensitivity plasma CRP levels were measured in duplicate by an in-house enzyme-linked immunosorbent assay (ELISA) based on purified protein and polyclonal anti-CRP antibodies (Dako, Glostrup, Denmark). Plasma IL-6 was measured in duplicate by a high-sensitivity ELISA (PeliKine Compact^TM^ ELISA; Sanquin, Amsterdam, The Netherlands), and TNF-α was assayed in duplicate using a high-sensitivity solid phase ELISA (Quantikine^®^ HS Human TNF-α Immunoassay; R&D systems Inc., Minneapolis, USA). Intra- and inter-assay coefficients of variation were 5% and 10% for CRP, 8% and 12% for IL-6, and 10% and 15% for TNF-α. To obtain normal distributions, the values of CRP, IL-6 and TNF-α were ln-transformed. An overall measure of basal inflammation indicative of more systemic inflammation was created by calculating a basal inflammatory index as the standardized sum of all three standardized ln-transformed basal markers.

*Innate immune response* of 17 cytokines was examined at baseline by ex vivo LPS stimulation of blood. Data collection for the LPS procedure was only conducted in the last year of the baseline assessment and therefore LPS-stimulated samples were only available in a (random) subset of 1242 NESDA participants. Of the 1078 MDD patients included in the current study, 454 patients had available data on LPS-stimulated markers. Patients with available data on these LPS-stimulated markers did not differ from the remainder of the study sample in terms of age, sex, education, depression severity and presence of anxious distress. Serial venous whole blood samples were obtained in one 7-ml heparin-coated tube (Greiner Bio-one, Monroe, North Carolina), of which 4.5 ml blood was stimulated by addition of LPS (10 ng/ml blood; *Escherichia coli*, Sigma, St. Louis, MO, USA) and these LPS-stimulated samples were laid flat and incubated at a slow rotation for 5–6 h at 37°C. Using a multi-analyte profile (Human CytokineMAP A v 1.0; Myriad RBM, Austin, USA), levels of granulocyte–macrophage colony-stimulating factor (GM-CSF), interferon (IFN)-γ, IL-2, IL-3, IL-4,IL-5, IL-6, IL-7, IL-8, IL-10, IL-18, monocyte chemotactic protein-1 (MCP-1), macrophage inflammatory protein (MIP)-1α, MIP-1β, matrix metalloproteinase-2 (MMP-2), TNF-α and TNF-β were determined. Too few values were obtained (valid *n *< 200) for several markers (GM-CSF, IL-3, IL-5 and IL-7) and were therefore excluded from analyses, leaving a total of 13 innate inflammatory markers. All cytokines, except MMP-2 and TNF-β, were ln-transformed to retrieve normal distribution patterns. To obtain a measure of overall innate production capacity, an LPS-stimulated inflammation index was calculated as the standardized sum of all 13 (normally distributed) standardized LPS-stimulated markers. Moderate to strong Pearson *r* correlations (0.34–0.88) between individual LPS-stimulated markers and LPS-stimulated index (Supplemental Table S5) supported the decision to combine all pro- and anti-inflammatory LPS-stimulated markers, as was also done in previous work^23^. In addition, the Pearson *r* correlation between the basal index and LPS-stimulated index was small (*r* = 0.16, *P* < 0.01; Table S5), supporting that these cover different aspects of the immune system.

### Covariates

Sociodemographic characteristics included age, sex and lab/study site (Amsterdam, Leiden, Groningen, Heerenveen). Lifestyle and health covariates included *smoking status* (never, former, current), *alcohol intake* (<1 drinks/week; 1–14/1–21 [female/male] drinks/week; >14/21 [female/male] drinks/week), *physical activity* (International Physical Activity Questionnaire^50^, expressed in 1000 metabolic equivalent [MET]-minutes/week), *number of self-reported chronic diseases under treatment* (heart disease, diabetes, stroke, lung disease, osteoarthritis, cancer, ulcer, intestinal problems, liver disease, epilepsy, thyroid gland disease) and *body mass index (BMI;* kg/m^2^). *Depression severity* was determined by the Quick Inventory of Depressive Symptomatology (QIDS)^51^ with 16 items. To avoid overlap with the anxious distress specifier, two overlapping IDS items (items 15 and 24) were excluded resulting in a total score ranging from 0 to 24. Past-month medication use was based on drug container inspection and classified according to the World Health Organization (WHO) Anatomical Therapeutic Chemical (ATC) classification system^52^. *Antidepressant medication use* included use of selective serotonin reuptake inhibitors (SSRI; ATC-code N06AB), tricyclic antidepressants (TCA; ATC-code N06AA) and other antidepressants (Other AD; ATC-codes N06AF/N06AX). Use of *anti-inflammatory medication* (M01A/M01B/A07EB/A07EC) and *statins* (C10AA/C10B) was also determined.

### Statistical analyses

First, baseline characteristics and unadjusted levels of basal and LPS-stimulated markers were described in the whole MDD sample (*N* = 1078), and compared between MDD patients with and without the specifier. Chi-square tests were used for categorical variables, independent *t*-tests for continuous variables, and Mann–Whitney *U*-tests for non-normally distributed variables. Second, adjusted associations between basal and LPS-stimulated markers with presence of the anxious distress specifier were analyzed using analyses of covariance. Associations were adjusted for site, age, sex, and lifestyle and health covariates. Cohen’s *d* ^53^ was calculated to estimate effect sizes. Finally, adjusted associations between the basal and LPS-stimulated indices with various dimensional anxiety indicators were analyzed using linear regression. We did not correct for use of anti-inflammatory medication or statins, as the prevalence of use was very low and non-significantly different across groups. In all adjusted analyses, the specifier and other anxiety indicators were used as independent variables, and inflammatory markers as dependent variables.

Several other covariates (depression severity, SSRI use, BMI) were not adjusted for in the primary analysis, but adjustment for these are reported in the Supplement (see Supplemental Tables S2 and S3). Correction for these factors may be considered an overcorrection given that patients with MDD meeting criteria for the anxious distress specifier already have a higher depression severity by definition (i.e. more symptoms are required). It has also been suggested that anxiety may be an epiphenomenon of the more severe forms of MDD, rather than a distinct phenomenon^9^. Antidepressant use is also likely to reflect depression severity, and prior analyses have shown only some restricted associations with LPS-stimulated inflammatory indicators^23^. It has been shown that BMI affects basal inflammation more strongly than innate production^23^. Adipose tissue produces cytokines and may lie on the same pathway as inflammation, which can have a mediating effect^20^. Therefore, correction for BMI may be considered an overcorrection. Statistical analysis was conducted with SPSS, version 22 (IBM Corp.: Armonk, New York), and all statistical tests were two-sided. Since we analyzed correlated measures that reflect one central concept, we corrected for multiple testing using the Benjamini–Yekutieli method^54,55^ that takes dependency of test statistics into account. We calculated an adjusted *P*-value of 0.012 correcting for the number of tests performed (*N* = 34). Results with *P*-values smaller than the adjusted threshold of 0.012 were considered significant.

## Results

The MDD sample (*N* = 1078) had a mean age of 40.9 (SD = 12.05) years, 67.3% were female, had a mean QIDS score of 10.8 (SD = 4.4) and 65.5% had a concurrent anxiety disorder. The anxious distress specifier was present in over half of the MDD patients (*n* = 585; 54.3%) (Table 1). In addition, presence of the anxious distress specifier poorly overlapped with presence of any comorbid DSM-IV-based anxiety disorder diagnosis (Cohen kappa = 0.09). Quite a few MDD persons with comorbid anxiety disorders (*n* = 253, 23.5%) do not fulfill the anxious distress specifier, and vice versa, quite some MDD persons with anxious distress (*n* = 132, 12.2%) do not fulfill criteria for a comorbid DSM-IV-based anxiety disorder, indicating that concepts are not directly comparable.

**Table 1:** Baseline characteristics in patients with current (past 6 months) MDD, and with and without the DSM-5 anxious distress specifier

	Current MDD	With anxious distress specifier	Without anxious distress specifier	*P* ^a^	*N*
	*N *= 1078	*N *= 585	*N* = 493		
Sociodemographic characteristics					
Age, years, mean ± SD	40.9 ± 12.05	41.3 ± 12.0	40.5 ± 12.1	0.288	1078
Sex, female, *n* (%)	725 (67.3)	390 (66.7)	335 (68.0)	0.654	1078
Education, years, mean ± SD	11.7 ± 3.2	11.2 ± 3.3	12.1 ± 3.1	<0.001	1078
Laboratory site,* n* (%)					
Amsterdam	391 (36.3)	214 (36.6)	177 (35.9)	0.163	1078
Leiden	394 (36.5)	223 (38.1)	171 (34.7)		
Groningen	205 (19.0)	99 (16.9)	106 (21.5)		
Emmen	64 (5.9)	39 (6.7)	25 (5.1)		
Heerenveen	24 (2.2)	10 (1.7)	14 (2.8)		
Clinical characteristics					
Depression severity, QIDS score, mean ± SD	10.8 ± 4.4	12.9 ± 3.8	8.3 ± 3.8	<0.001	1078
Antidepressant medication use, *n* (%)					
Tricyclic antidepressant	44 (4.1)	23 (3.9)	21 (4.3)	0.786	1078
Selective serotonin reuptake inhibitor	316 (29.3)	188 (32.1)	128 (26.0)	0.027	1078
Other antidepressant	120 (11.1)	70 (12.0)	50 (10.1)	0.343	1078
Lifestyle and health factors					
Smoking status, current smoker, *n* (%)	482 (44.7)	268 (45.8)	214 (43.4)	0.731	1078
Alcohol intake, *n* (%)					
<1 drink a week	438 (40.6)	261 (44.6)	177 (35.9)	0.003	1078
1–14/1–21 (women/men) drinks a week	598 (55.5)	297 (50.8)	301 (61.1)		
>14/>21 (women/men) drinks a week	42 (3.9)	27 (4.6)	15 (3.0)		
Physical activity (1000 MET-min), mean ± SD	3.5 ± 3.1	3.5 ± 3.1	3.6 ± 3.2	0.318	1078
Body mass index (kg/m^2^), mean ± SD	25.9 ± 5.4	26.2 ± 5.5	25.5 ± 5.3	0.028	1078
Number of chronic diseases, *n* (%)					
0	579 (53.7)	294 (50.3)	285 (57.8)	0.010	1078
1	328 (30.4)	182 (31.1)	146 (29.6)		
≥2	171 (15.9)	109 (18.6)	62 (12.6)		
Systemic anti-inflammatory medication, *n* (%)	52 (4.8)	29 (5.0)	23 (4.7)	0.824	1078
Statin use, *n* (%)	76 (7.1)	41 (7.0)	35 (7.1)	0.954	1078
Anxiety constructs					
Any current anxiety disorder, n (%)	706 (65.5)	453 (77.4)	253 (51.3)	<0.001	1078
Number of anxiety disorders, median (IQR)	1.0 (0.0–2.0)	1.0 (1.0–2.0)	1.0 (0.0–1.0)	<0.001	1078
IDS anxiety arousal subscale, mean ± SD	16.4 ± 4.2	18.7 ± 3.5	13.7 ± 3.1	<0.001	1077
Beck Anxiety Inventory, mean ± SD	18.1 ± 11.2	24.5 ± 10.4	10.5 ± 6.6	<0.001	1078
Fear Questionnaire, mean ± SD	33.7 ± 21.6	41.2 ± 22.0	24.8 ± 17.4	<0.001	1078
MASQ anxious arousal subscale, mean ± SD	18.9 ± 6.9	21.9 ± 7.1	15.5 ± 4.7	<0.001	894
Anxiety Sensitivity Index, mean ± SD	17.3 ± 10.3	20.8 ± 10.8	13.4 ± 8.0	<0.001	910
Penn State Worry Questionnaire, mean ± SD	38.2 ± 9.9	41.7 ± 8.8	34.3 ± 9.6	<0.001	906

*IDS* Inventory of Depressive Symptomatology, *MASQ*Mood and Anxiety Symptoms Questionnaire, *MDD* major depressive disorder, *MET* metabolic equivalent, *QIDS*, Quick Inventory of Depressive Symptomatology. ^a^For *P-*value: *t*-tests were used for continuous variables; Chi-square analyses were used for dichotomous variables; Mann–Whitney *U*-tests were used for non-normal distributed variables

Sociodemographics were comparable between MDD patients with and without the anxious distress specifier, with the exception of one year less education in patients meeting criteria for the specifier (*P* < 0.001). In addition, patients with the specifier, compared to those without, had a higher depression severity (*P* < 0.001) and were more likely to use SSRIs (*P* = 0.016). Patients with the specifier reported more alcohol intake (*P* = 0.002), had a higher BMI (*P* = 0.028), and reported more chronic diseases (*P* = 0.012). As expected, all anxiety indicators were significantly higher in the MDD patients with the specifier (*P* < 0.001) (Table 1).

Unadjusted levels of all basal inflammatory markers did not significantly differ between MDD patients with and without the specifier (Table 2). In contrast, several unadjusted LPS-stimulated inflammation levels (MMP-2 and LPS-stimulated index) were significantly higher in patients with than without the specifier (Table 2).

**Table 2:** Unadjusted levels of basal and LPS-stimulated inflammatory markers in patients with current (past 6 months) MDD, and with and without the anxious distress specifier

	Current MDD	With anxious distress specifier	Without anxious distress specifier	*P* ^a^	*N*
	*N* = 1078	*N* = 585	*N* = 493		
Basal inflammatory markers					
CRP (mg/l), med (IQR)	1.18 (0.54–2.93)	1.16 (0.53–2.81)	1.23 (0.56–3.01)	0.844	1011
IL-6 (pg/ml), med (IQR)	0.81 (0.51–1.33)	0.81 (0.52–1.29)	0.77 (0.50–1.35)	0.436	1076
TNF-α (pg/ml), med (IQR)	0.80 (0.60–1.10)	0.80 (0.60–1.10)	0.80 (0.60–1.15)	0.886	1069
Basal inflammation index, mean ± SD	0.06 ± 1.0	0.02 ± 0.98	−0.02 ± 1.02	0.572	1003
LPS-stimulated inflammatory markers					
IFN-γ (pg/ml), med (IQR)	10.20 (7.31–14.00)	10.50 (8.00–14.03)	9.82 (6.22–14.00)	0.065	454
IL-2 (pg/ml), med (IQR)	9.24 (6.39–13.70)	9.51 (6.79–13.88)	8.59 (5.78–12.60)	0.037	454
IL-4 (pg/ml), med (IQR)	9.03 (4.23–15.23)	9.03 (4.23–15.00)	9.14 (4.23–15.90)	0.958	454
IL-6 (ng/ml), med (IQR)	26.70 (18.05–34.80)	27.60 (19.90–37.20)	25.05 (16.38–33.55)	0.015	453
IL-8 (ng/ml), med (IQR)	11.20 (7.90–16.70)	11.40 (8.16–17.40)	10.60 (7.47–15.98)	0.170	453
IL-10 (pg/ml), med (IQR)	203.50 (114.75–380.50)	205.50 (121.25–409.25)	200.00 (108.75–339.25)	0.131	454
IL-18 (pg/ml), med (IQR)	254.00 (209.00–314.25)	260.00 (214.75–316.50)	249.50 (202.00–313.75)	0.234	454
MCP-1 (ng/ml), med (IQR)	1.60 (1.05–2.33)	1.70 (1.17–2.46)	1.57 (0.91–2.22)	0.020	454
MIP-1α (ng/ml), med (IQR)	18.80 (12.70–25.75)	20.00 (13.60–26.65)	17.05 (11.70–23.83)	0.014	453
MIP-1β (ng/ml), med (IQR)	240.00 (169.00–316.00)	244.00 (170.50–316.00)	228.50 (166.50–316.00)	0.143	453
MMP-2 (ng/ml), mean ± SD	74.12 ± 18.75	76.74 ± 17.73	70.67 ± 19.54	0.001*	454
TNF-α (ng/ml), med (IQR)	2.77 (1.87–4.01)	2.90 (1.96–4.27)	2.67 (1.69–3.77)	0.022	453
TNF-β (pg/ml), mean ± SD	328.03 ± 135.38	339.74 ± 124.26	312.62 ± 147.68	0.039	454
LPS inflammation index, mean ± SD	0.12 ± 0.85	0.23 ± 0.70	−0.03 ± 1.00	0.002*	453

*CRP*C-reactive protein;* IFN*interferon; *IL*interleukin*; LPS* lipopolysaccharide; MCP monocyte chemotactic protein, *MDD* major depressive disorder; *med* median; *MIP* macrophage inflammatory protein; *MMP* matrix metalloproteinase; *TNF*tumor necrosis factor. ^a^For *P-*value: *t*-tests were used for continuous variables; Mann–Whitney *U*-tests were used for non-normal distributed variables. *Results survive the Benjamini–Yekutieli correction for multiple comparisons threshold of <0.012

**Fig. 1:**
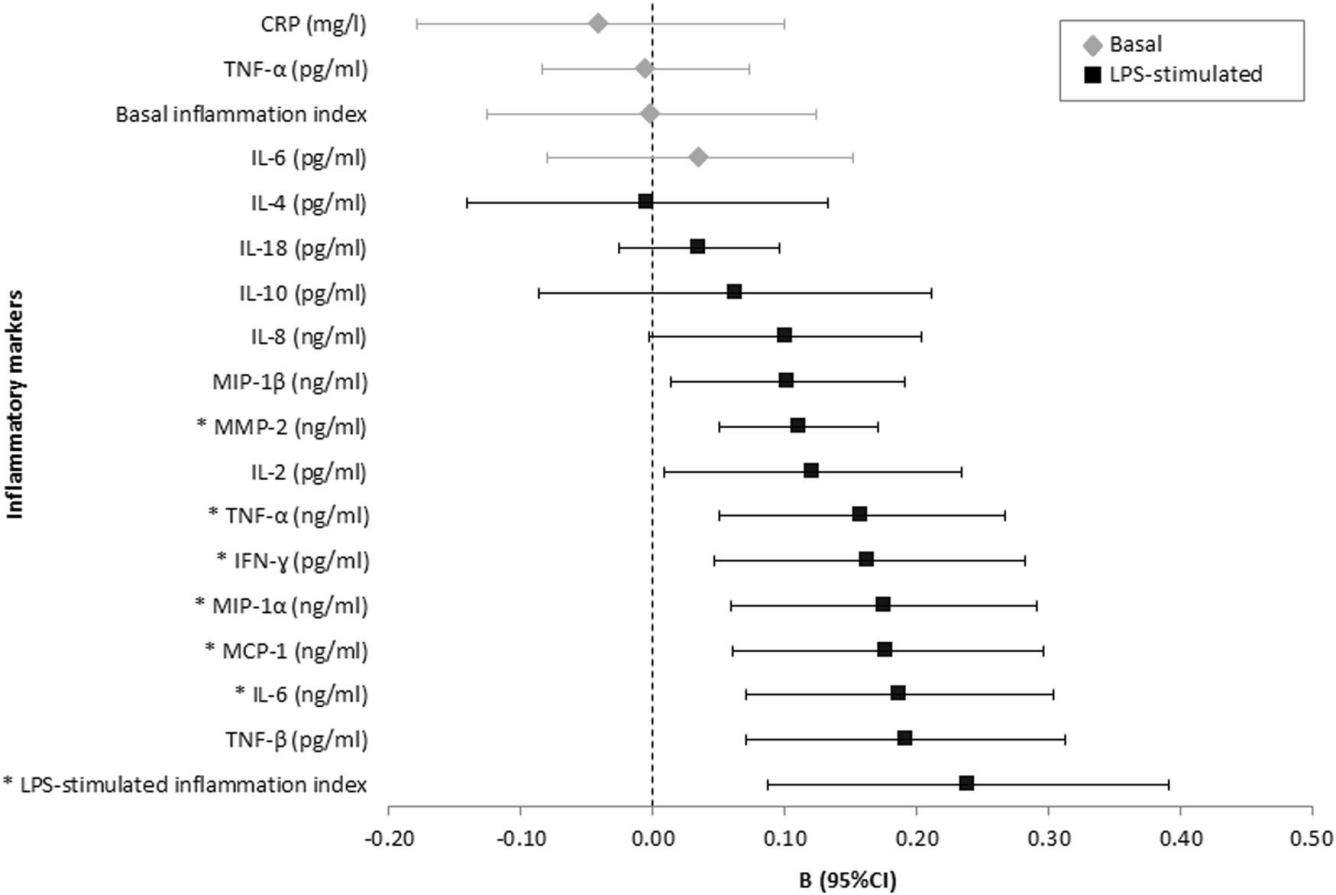
Adjusted difference in basal and innate inflammatory markers between MDD patients with versus without (ref) the DSM-5 anxious distress specifier. *Results survive the Benjamini–Yekutieli correction for multiple comparisons threshold of <0.012, adjusted regression coefficients and error bars (95% CI) of ln-transformed inflammatory markers (MMP-2 and TNF-β were also ln-transformed for easy comparison). CRP C-reactive protein, IFN interferon, IL interleukin, LPS lipopolysaccharide, MCP, monocyte chemotactic protein, MDD major depressive disorder, MIP, macrophage inflammatory protein, MMP matrix metalloproteinase, TNF tumor necrosis factor. Based on analyses of covariance. Adjusted for site, age, sex, smoking status, alcohol intake, physical activity and number of chronic diseases under treatment

Adjusted analyses were adjusted for sociodemographics (i.e. site, age, sex) and lifestyle and health covariates (i.e. smoking status, alcohol intake, physical activity, number of self-reported chronic diseases under treatment). We determined the specific effects of all selected covariates on the basal and LPS-stimulated inflammation index in one multiple linear regression model for each index. The basal inflammation index was associated with smoking status (*β* = 0.09, *P* = 0.001), QIDS severity (*β* = 0.13, *P* < 0.001) and BMI (*β* = 0.36, *P* < 0.001), whereas the LPS-stimulated inflammation index was associated with sex (*β* = −0.16, *P* < 0.001), site (*β* = −0.23, *P* < 0.001) and smoking status (*β* = 0.14, *P* = 0.003). The adjusted difference in levels of basal markers remained non-significant between MDD patients with and without anxious distress (Fig. 1; Table S1). Interestingly, for the innate production capacity, effects of anxious distress appeared stronger after adjustment for sociodemographics and important lifestyle and health covariates with small to medium effect sizes. A higher number of adjusted (6 out of 13) than unadjusted (1 out of 13) levels of the LPS-stimulated markers (i.e. IFN-γ, IL-6, MCP-1, MIP-1α, MMP-2, TNF-α) as well as the LPS-stimulated inflammation index were significantly higher in MDD patients with anxious distress (Fig. 1; Table S1). Adjusted levels of three out of these six markers remained significantly higher in patients with anxious distress after adjustment for depression severity (QIDS score) (Table S2). After adjustment for SSRI use and BMI, all six LPS-stimulated markers and the LPS-stimulated index remained significantly higher (all *P*-values <0.012) and effect sizes were comparable after additional adjustment (Table S2). We specifically aimed to examine within a large sample of MDD whether anxious distress was associated with differential inflammation. No healthy control group is required to answer this particular research question. Nevertheless, we ran additional analyses with the inclusion of healthy controls to place the main findings of MDD with anxious distress in a broader, conceptual framework and to determine whether increased innate cytokine production capacity, as compared to healthy controls, was indeed the most dysregulated in MDD with anxious distress and that MDD without anxious distress differed less in this respect. Therefore, we not only examined whether innate cytokine production capacity was increased in MDD with anxious distress when compared to MDD without anxious distress, but also examined whether innate cytokine production capacity was increased in MDD with anxious distress when compared to healthy controls. Adjusted analyses were repeated for the significant associations with the 6 out of 13 LPS-stimulated markers and LPS-stimulated index with the addition of a healthy control group. NESDA participants without a lifetime depressive and anxiety disorder and who had available data on LPS-stimulated markers were included in the control group (*N *= 297). All LPS-stimulated levels and index remained significant between MDD patients with anxious distress and controls, with the exception of IFN-ɣ (*P* = 0.144) (Figure S1A). In contrast, all LPS-stimulated levels and index were non-significant between MDD patients without anxious distress and controls (Figure S1B).

When examining associations between the inflammation indices with various anxiety indicators within the MDD sample, a positive association was found between the basal inflammation index and BAI score (*P* = 0.011) of modest strength after adjustment for sociodemographics and lifestyle and health factors (Table 3). For the seven other anxiety indicators, no associations with the basal index were found. In contrast, a higher number of positive associations were found between the LPS-stimulated inflammation index and various indicators of high anxiety: the anxious distress specifier score (*P* = 0.001), the BAI (*P* < 0.001) and the MASQ-AA (*P* = 0.007) (Table 3). Effects for some anxiety indicators became non-significant after adjustment for QIDS score and only MASQ-AA became marginally significant (*P* = 0.017) after adjustment for SSRI use, but all indicators remained significant when adjusting for BMI (all *P*-values <0.012) (Table S3).

**Table 3:** Adjusted associations between basal inflammation index and LPS-stimulated inflammation index with various dimensional anxiety constructs in patients with current (past 6 months) MDD

	Basal inflammation index			LPS-stimulated inflammation index		
	*β*	*P* ^a^	*R* ^2^	*β*	*P* ^a^	*R* ^2^
Anxious distress specifier score	0.04	0.194	0.055	0.15	0.001*	0.135
Number of anxiety disorders	0.03	0.363	0.055	0.11	0.021	0.123
IDS anxiety arousal subscale	0.07	0.021	0.059	0.10	0.026	0.122
Beck Anxiety Inventory	0.08	0.011*	0.060	0.19	<0.001*	0.146
Fear Questionnaire	0.06	0.051	0.058	0.09	0.063	0.119
MASQ anxious arousal scale	0.04	0.256	0.055	0.14	0.007*	0.156
Anxiety Sensitivity Index	0.01	0.867	0.054	0.09	0.061	0.148
Penn State Worry Questionnaire	0.01	0.873	0.054	0.09	0.070	0.146

*IDS* Inventory of Depressive Symptomatology, *LPS* lipopolysaccharide, *MASQ* Mood and Anxiety Symptoms Questionnaire; *MDD*major depressive disorder. ^a^Based on linear regression analyses. Adjusted for site, age, sex, smoking status, alcohol intake, physical activity and number of chronic diseases under treatment. *Results survive the Benjamini–Yekutieli correction for multiple comparisons threshold of <0.012

In a post-hoc analysis we evaluated the correlation between the anxiety measures to see whether the dimensional anxiety scales with more somatic items were strongly correlated (Table S4). Correlations between the somatic-oriented IDS-AA, BAI and MASQ-AA were strong (0.63–0.74), whereas these were moderate (0.35–0.55) between the cognitive-oriented anxiety indicators (FQ, ASI, PSWQ) (Table S4).

## Discussion

This study is the first to examine whether anxious distress and related anxiety features are associated with dysregulated basal inflammatory markers and innate cytokine production capacity within a large MDD sample. Our most important finding is that higher innate production capacity, rather than higher basal inflammation, was associated with anxious distress in a large MDD sample. Anxious distress in MDD patients (present in 54.3%) was not associated with higher basal inflammation, but a modest positive association was found for BAI score. However, anxious distress was associated with higher LPS-stimulated inflammation levels (i.e. IFN-γ, IL-6, MCP-1, MIP-1α, MMP-2, TNF-α and LPS-stimulated inflammation index). Other indicators of high anxiety (i.e. anxious distress specifier score, BAI and MASQ-AA scores) confirmed an increased innate cytokine production capacity in MDD patients scoring high on the anxiety dimension.

The results support the hypothesis that patients with the anxious distress specifier may have different underlying biological profiles than patients without this specifier, which could contribute to earlier findings showing that the anxious distress specifier predicts poorer course and treatment outcomes^2,10^. Our study supports the recent suggestion of the specific involvement of inflammation in the development of anxious depression^36^. Anxious depression has distinct neurobiological correlates that separates it from MDD alone^11–16^, and our results further support and extend this in light of inflammation.

Anxious distress within MDD patients was not associated with higher basal inflammation. Possibly, this may be due to the fact that basal inflammation levels are usually low and have high within-person variability, making it harder to detect immune dysregulations^23^. Also, basal inflammation levels are known to be rather strongly influenced by lifestyle and (somatic) disease factors^20,23^. However, even the unadjusted levels of basal inflammation did not differ between MDD patients with and without anxious distress. In contrast to our findings, one study showed that MDD patients with moderate-severe to severe anxious distress tended to have higher CRP levels than those with mild to moderate anxious distress^35^. However, the difference in CRP levels was non-significant after adjustment and no other basal inflammation markers were assessed in that study. Serum concentration of CRP is a complicated diagnostic biomarker of depression. While synthesis of the acute-phase protein CRP in the liver is mainly induced by IL-6 and IL-1, a range of other factors might induce its synthesis as well^56^. Although previous studies have indicated elevated CRP in depressed patients compared with healthy controls^20,57^, interestingly, it has been shown that the rs1205(G/A) genetic polymorphism of the CRP gene is associated with both lower CRP levels and greater risk of depression^58,59^. Some of this complexity likely arises from the biological heterogeneity in depression risk and resilience factors within the MDD population. Cytokines on the other hand are more specific cell-based measures of inflammation rather than the non-specific inflammation marker CRP, and it has been shown that relevant genetic variants of specific cytokines were associated with increased risks of depression development^59^. This could explain why we find increased levels of several cytokines in persons with MDD and anxiety but not of CRP. A modest positive association was found for higher basal inflammation and BAI score, possibly because this anxiety indicator has a greater focus on somatic, rather than cognitive, anxiety symptoms.

Interestingly, for innate production capacity, associations with the anxious distress specifier became stronger after adjustment for several important lifestyle and health factors. Also, all findings remained significant after additional adjustment for BMI and SSRI use, supporting the suggestion that innate production capacity is not highly influenced by lifestyle and health status, in line with previous findings^23^. After additional adjustment for depression severity, some of the positive associations between presence of the specifier and higher levels of LPS-stimulated inflammatory markers and index in MDD patients diminished to non-significance. In addition, although the value of adjusting for SSRI use, BMI and overall depression severity is unclear and can be considered as overcorrection, we nevertheless corrected for these factors in additional analyses, showing that main findings were not different after adjustment for these extra factors. Moreover, these findings were extra confirmed when analyses were repeated with the addition of a healthy control group. Thus, it seems that the link between innate production capacity and anxious distress in MDD is not mainly driven by depression severity but likely relies on anxiety aspects.

Higher innate production was positively associated with anxiety indicators (i.e. BAI and MASQ-AA scores) with a greater focus on somatic anxiety symptoms, and these indicators were strongly correlated. This may imply that the association between anxious distress in MDD and higher innate production is mainly driven by somatic anxiety symptoms. This is in line with earlier research where somatic, but not cognitive, symptoms of anxiety and depression were associated with basal inflammation^60^. Moreover, somatic anxiety symptoms may reflect the hypervigilance characteristic of anxiety disorders—which was suggested to be part of the ‘pathogen host defence’ hypothesis^17^, thereby the results support this hypothesis. It is also possible that somatic anxiety symptoms are associated with increased sympathetic and decreased parasympathetic autonomic activity^61^, since the autonomic nervous system is associated with higher inflammation levels^18,62,63^. Another possibility for the anxiety-inflammation link is that inflammatory markers in the brain affect metabolic and molecular pathways that influence neurotransmitter systems (e.g. monoamines and glutamate) which ultimately affect neurocircuits regulating anxiety^17,34^. It is also possible that anxiety, reflected as psychological stress, may induce inflammasome pathways leading to inflammation and ultimately to depression^17,32^. The innate inflammatory response is under strong genetic control^39^ with heritability estimates ranging from 53 to 86% in non-diseased populations^39,64^. It may be possible that the anxious subtype of MDD has shared genetics with the innate production capacity. Future research should further examine this genetic link. Another possible explanation for the found associations between higher LPS-stimulated levels and anxious distress in MDD patients may be that the inflammatory signals become amplified after ex vivo LPS stimulation making it easier to find associations, rather than that basal inflammation and innate production capacity are truly different immune concepts.

With regard to different subtypes of MDD and inflammatory dysregulations, it has been shown in some studies, but not all, that elevated basal inflammation levels were more common in atypical than in melancholic depression and healthy controls^65^. Our group previously found that an atypical and melancholic symptoms subscale were to the same extent associated with increased innate production capacity, but these associations were no longer significant after health adjustment^23^. However, our findings show that after lifestyle and health adjustment, anxious distress in MDD patients is associated with increased innate production capacity, but not with basal inflammation. Post-hoc analyses showed that when repeating the analyses while adjusting for both the atypical^66^ and melancholic^67^ symptom subscale together in one model, the significant associations between 6 out of 13 LPS-stimulated markers and LPS-stimulated index with the anxious distress specifier remained significant. This clearly indicates that these are unrelated findings.

The anxious distress specifier appears to identify a clinically relevant group of patients who may have a poor course and poor treatment response^2,10^, and which seems to involve a stronger innate cytokine production capacity that separates it from MDD alone. It has been shown in patients with high baseline inflammatory biomarkers that anti-inflammatory treatment with TNF-α antagonists may improve depressive symptoms^68^. A meta-analysis by Kohler and colleagues (2014)^69^ suggested the positive antidepressant effects of anti-inflammatory treatment such as nonsteroidal anti-inflammatory drugs and cyclooxygenase-2 inhibitors in depression but this finding was uncertain because of high heterogeneity. This underscores the need to identify depressive subgroups with high inflammation that may benefit from anti-inflammatory treatments. Our study contributes to the reduction in MDD heterogeneity, and supports the idea that the anxious MDD subgroup may specifically benefit from anti-inflammatory agents or new antidepressants with a target on inflammation.

Several limitations should be noted. The specifier used for this study was constructed by self-reported proxy items instead of clinician-based assessments^2^. However, results from our previous study demonstrated that our specifier reflected a valid conceptual assessment for anxious depression with significant discriminant performance and convergent and predictive validity^2^. We selected patients who had an MDD diagnosis with a 6-month recency in order to obtain a large enough sample, which has also been utilized in our previous work examining the longitudinal course and treatment impact of the anxious distress specifier^2,10^. Of our MDD sample, almost all (95%) had truly evidence of recent symptoms as indicated by either the presence of MDD with a 1-month recency or an IDS score ≥14, indicating clinically relevant depression severity symptoms. Similar results were obtained when removing the 5% not meeting this currency criteria, showing that inclusion of these patients did not impact our results. While we adjusted for number of chronic somatic diseases under treatment in our primary analyses as a measure of overall burden of somatic disease, we did not adjust for a specific type of somatic disease. However, we determined the independent effects of the various somatic diseases, and found that only chronic non-specific lung diseases (i.e. asthma, chronic bronchitis and pulmonary emphysema) had a significant effect on the LPS-stimulated inflammatory index (*P* = 0.047). When adjusting for all specific types of somatic diseases, this did not change our conclusions which further supports the decision to adjust for number of somatic diseases. This study used cross-sectional data and therefore no causal inferences can be made. The study also has a number of important strengths. Due to the large sample size it was possible to adequately adjust for important covariates. In addition to more traditional dimensional anxiety indicators, we also examined the new DSM-5 anxious distress specifier. Furthermore, we not only examined basal inflammatory markers, but also LPS-stimulated inflammatory markers which give more insight into the functioning and profile of the immune system which is under strong genetic control. That the DSM-5 anxious distress specifier exhibited one of the strongest associations with LPS-stimulated inflammation index suggests that these five simple questions are enough to select a group of MDD patients who are more homogeneous biologically than the MDD population as a whole. With efforts to develop treatments for depression that target immune dysregulation, it will be of interest to see whether the DSM-5 specifier could serve as a means for selecting patients suitable for such treatments.

Our study within a large MDD sample clearly indicates that those MDD patients with anxious distress showed an increased innate cytokine production capacity but no higher basal inflammation. These results provide new insight into the pathophysiology of MDD with concurrent anxiety features, and contribute to the idea that the anxious subtype of MDD is an individual subtype that involves specific pathophysiology.

## Electronic supplementary material

Supplemental Tables

Supplemental Figure S1
