# Supplementary material for: The role of anxious distress in immune dysregulation in patients with major depressive disorder
Source: Transl Psychiatry. 2017 Dec 8;7:1268. doi: 10.1038/s41398-017-0016-3 (PMC5802575; doi:10.1038/s41398-017-0016-3)
Supplement: Supplementary file 1 — Supplemental Tables [file 41398_2017_16_MOESM1_ESM.docx]

**Supplementary tables**

**Table S1.** Adjusted associations between basal and LPS-stimulated inflammatory markers with presence of the DSM-5 anxious distress specifier in patients with current (past 6 months) MDD.

|  | **Presence of the anxious distress specifier** | | | | |  | |
| --- | --- | --- | --- | --- | --- | --- | --- |
|  | **Yes** | | **No** | |  |  | |
|  | **Mean** | **95% CI** | **Mean** | **95% CI** | ***P*^a^** | ***d*** | |
| **Basal inflammatory markers** |  |  |  |  |  |  | |
| CRP (mg l^-1^)^b^ | 1.16 | 1.06-1.28 | 1.21 | 1.09-1.34 | 0.578 | -0.004 |  |
| IL-6 (pg ml^-1^)^b^ | 0.81 | 0.75-0.87 | 0.78 | 0.71-0.85 | 0.539 | 0.004 |  |
| TNF-α (pg ml^-1^)^b^ | 0.86 | 0.81-0.91 | 0.86 | 0.82-0.92 | 0.898 | -0.008 |  |
| Basal inflammation index | 0.07 | -0.02-0.15 | 0.07 | -0.03-0.16 | 0.990 | 0.000 |  |
| **LPS-stimulated inflammatory markers** |  |  |  |  |  |  |  |
| IFN-ɣ (pg/ml)^b^ | 10.78 | 9.99-11.63 | 9.14 | 8.38-9.98 | 0.006* | 0.262 |  |
| IL-2 (pg/ml)^b^ | 9.21 | 8.55-9.90 | 8.15 | 7.49-8.87 | 0.035 | 0.204 |  |
| IL-4 (pg/ml)^b^ | 8.67 | 7.93-9.48 | 8.71 | 7.85-9.64 | 0.954 | -0.006 |  |
| IL-6 (ng/ml)^b^ | 25.51 | 23.64-27.52 | 21.16 | 19.38-23.08 | 0.002* | 0.300 |  |
| IL-8 (ng/ml)^b^ | 11.63 | 10.87-12.44 | 10.52 | 9.74-11.37 | 0.056 | 0.185 |  |
| IL-10 (pg/ml)^b^ | 213.36 | 193.64-235.10 | 200.34 | 179.29-224.08 | 0.408 | 0.079 |  |
| IL-18 (pg/ml)^b^ | 261.13 | 250.89-271.51 | 252.14 | 240.81-264.01 | 0.262 | 0.109 |  |
| MCP-1 (ng/ml)^b^ | 1.63 | 1.51-1.76 | 1.36 | 1.25-1.49 | 0.003* | 0.283 |  |
| MIP-1α (ng/ml)^b^ | 18.21 | 16.89-19.65 | 15.27 | 14.00-16.66 | 0.003* | 0.283 |  |
| MIP-1β (ng/ml)^b^ | 236.28 | 222.96-250.13 | 213.15 | 199.54-227.69 | 0.023 | 0.219 |  |
| MMP-2 (ng/ml) | 76.77 | 74.73-79.17 | 70.40 | 67.86-72.95 | <0.001* | 0.337 |  |
| TNF- α (ng/ml)^b^ | 2.81 | 2.62-3.02 | 2.40 | 2.21-2.60 | 0.004* | 0.276 |  |
| TNF- β (pg/ml) | 339.19 | 324.41-357.31 | 311.15 | 292.26-330.05 | 0.044 | 0.192 |  |
| LPS-stimulated inflammation index | 0.22 | 0.12-0.32 | -0.02 | -0.13-0.09 | 0.002* | 0.296 |  |

Abbreviations: LPS, lipopolysaccharide; MDD, Major Depressive Disorder; CRP, C-reactive protein; IL, interleukin; TNF, tumor necrosis factor; IFN, interferon; MCP, monocyte chemotactic protein; MIP, macrophage inflammatory protein; MMP, matrix metalloproteinase.
^a^ Based on analyses of covariance. ^b^ Ln-transformed factors presented back-transformed. Adjusted for site, age, sex, smoking status, alcohol intake, physical activity and number of chronic diseases under treatment. * Results survive the Benjamini-Yekutieli correction for multiple comparisons threshold of <0.012.

**Table S2.** Adjusted associations between LPS-stimulated inflammatory markers and presence of the DSM-5 anxious distress specifier in patients with current (past 6 months) MDD, additionally adjusted for QIDS score, BMI and SSRI use.

|  | **Presence of the anxious distress specifier** | | | | |  | |
| --- | --- | --- | --- | --- | --- | --- | --- |
|  | **Yes** | | **No** | |  |  | |
|  | **Mean** | **95% CI** | **Mean** | **95% CI** | ***P*^a^** | ***d*** | |
| IFN-ɣ (pg/ml)^b^ |  |  |  |  |  |  | |
| QIDS score adjusted^c^ | 10.94 | 10.07-11.88 | 8.97 | 8.14-9.89 | 0.005* | 0.291 |  |
| BMI adjusted^d^ | 10.77 | 9.98-11.64 | 9.15 | 8.38-9.99 | 0.006* | 0.259 |  |
| SSRI use adjusted^e^ | 10.76 | 9.96-11.61 | 9.17 | 8.40-10.01 | 0.008* | 0.254 |  |
| IL-2 (pg/ml)^b^ |  |  |  |  |  |  |  |
| QIDS score adjusted^c^ | 9.18 | 8.47-9.93 | 8.18 | 7.45-8.98 | 0.090 | 0.175 |  |
| BMI adjusted^d^ | 9.20 | 8.55-9.90 | 8.15 | 7.49-8.87 | 0.035 | 0.202 |  |
| SSRI use adjusted^e^ | 9.18 | 8.53-9.88 | 8.17 | 7.52-8.90 | 0.045 | 0.194 |  |
| IL-4 (pg/ml)^b^ |  |  |  |  |  |  |  |
| QIDS score adjusted^c^ | 8.28 | 7.53-9.12 | 9.24 | 8.26-10.34 | 0.182 | -0.139 |  |
| BMI adjusted^d^ | 8.67 | 7.93-9.48 | 8.71 | 7.85-9.64 | 0.953 | -0.006 |  |
| SSRI use adjusted^e^ | 8.65 | 7.92-9.46 | 8.72 | 7.87-9.67 | 0.909 | -0.011 |  |
| IL-6 (ng/ml)^b^ |  |  |  |  |  |  |  |
| QIDS score adjusted^c^ | 25.48 | 23.48-27.66 | 21.16 | 19.22-23.31 | 0.008* | 0.273 |  |
| BMI adjusted^d^ | 25.48 | 23.62-27.49 | 21.18 | 19.41-23.10 | 0.002* | 0.302 |  |
| SSRI use adjusted^e^ | 25.38 | 23.52-27.39 | 21.26 | 19.49-23.22 | 0.003* | 0.284 |  |
| IL-8 (ng/ml)^b^ |  |  |  |  |  |  |  |
| QIDS score adjusted^c^ | 11.29 | 10.50-12.15 | 10.94 | 10.04-11.92 | 0.610 | 0.053 |  |
| BMI adjusted^d^ | 11.62 | 10.87-12.43 | 10.53 | 9.75-11.37 | 0.061 | 0.181 |  |
| SSRI use adjusted^e^ | 11.61 | 10.86-12.43 | 10.54 | 9.75-11.39 | 0.067 | 0.175 |  |
| IL-10 (pg/ml)^b^ |  |  |  |  |  |  |  |
| QIDS score adjusted^c^ | 215.72 | 194.03-293.61 | 197.75 | 174.69-223.63 | 0.333 | 0.099 |  |
| BMI adjusted^d^ | 213.36 | 193.45-235.10 | 200.54 | 179.29-224.30 | 0.419 | 0.078 |  |
| SSRI use adjusted^e^ | 213.15 | 193.25-234.86 | 200.74 | 179.47-224.53 | 0.433 | 0.075 |  |
| IL-18 (pg/ml)^b^ |  |  |  |  |  |  |  |
| QIDS score adjusted^c^ | 257.75 | 246.90-269.08 | 256.47 | 243.96-269.62 | 0.896 | 0.014 |  |
| BMI adjusted^d^ | 260.86 | 250.89-271.51 | 252.40 | 241.05-264.01 | 0.280 | 0.102 |  |
| SSRI use adjusted^e^ | 260.34 | 250.13-270.97 | 252.90 | 241.77-264.81 | 0.355 | 0.090 |  |
| MCP-1 (ng/ml)^b^ |  |  |  |  |  |  |  |
| QIDS score adjusted^c^ | 1.56 | 1.44-1.70 | 1.44 | 1.31-1.59 | 0.258 | 0.116 |  |
| BMI adjusted^d^ | 1.63 | 1.51-1.76 | 1.37 | 1.25-1.49 | 0.003* | 0.283 |  |
| SSRI use adjusted^e^ | 1.63 | 1.51-1.76 | 1.37 | 1.25-1.50 | 0.005* | 0.272 |  |
| MIP-1α (ng/ml)^b^ |  |  |  |  |  |  |  |
| QIDS score adjusted^c^ | 17.99 | 16.58-19.53 | 15.52 | 14.10-17.08 | 0.034 | 0.218 |  |
| BMI adjusted^d^ | 18.19 | 16.88-19.63 | 15.29 | 14.03-16.68 | 0.003* | 0.284 |  |
| SSRI use adjusted^e^ | 18.16 | 16.84-19.59 | 15.33 | 14.06-16.73 | 0.004* | 0.271 |  |
| MIP-1β (ng/ml)^b^ |  |  |  |  |  |  |  |
| QIDS score adjusted^c^ | 233.46 | 219.42-248.64 | 216.37 | 201.14-232.76 | 0.150 | 0.147 |  |
| BMI adjusted^d^ | 236.04 | 222.74-250.13 | 213.36 | 199.74-227.92 | 0.025 | 0.215 |  |
| SSRI use adjusted^e^ | 235.57 | 222.29-249.64 | 214.01 | 200.14-228.61 | 0.034 | 0.204 |  |
| MMP-2 (ng/ml) |  |  |  |  |  |  |  |
| QIDS score adjusted^c^ | 76.17 | 73.77-78.65 | 71.44 | 68.62-74.25 | 0.021 | 0.238 |  |
| BMI adjusted^d^ | 76.74 | 74.53-78.96 | 70.68 | 68.13-73.22 | <0.001* | 0.334 |  |
| SSRI use adjusted^e^ | 76.61 | 74.40-78.82 | 70.85 | 68.31-73.40 | 0.001* | 0.317 |  |
| TNF- α (ng/ml)^b^ |  |  |  |  |  |  |  |
| QIDS score adjusted^c^ | 2.85 | 2.64-3.08 | 2.35 | 2.15-2.57 | 0.003* | 0.301 |  |
| BMI adjusted^d^ | 2.81 | 2.62-3.02 | 2.40 | 2.21-2.60 | 0.004* | 0.276 |  |
| SSRI use adjusted^e^ | 2.81 | 2.61-3.01 | 2.40 | 2.21-2.60 | 0.005* | 0.268 |  |
| TNF- β (pg/ml) |  |  |  |  |  |  |  |
| QIDS score adjusted^c^ | 338.61 | 320.84-356.38 | 314.11 | 293.22-335.00 | 0.106 | 0.167 |  |
| BMI adjusted^d^ | 339.10 | 322.69-355.52 | 313.46 | 294.59-332.34 | 0.046 | 0.191 |  |
| SSRI use adjusted^e^ | 338.55 | 322.12-354.98 | 314.19 | 295.29-333.10 | 0.059 | 0.181 |  |
| LPS-stimulated inflammation index |  |  |  |  |  |  |  |
| QIDS score adjusted^c^ | 0.20 | 0.09-0.30 | 0.01 | -0.12-0.14 | 0.041 | 0.209 |  |
| BMI adjusted^d^ | 0.22 | 0.12-0.32 | -0.02 | -0.13-0.10 | 0.002* | 0.293 |  |
| SSRI use adjusted^e^ | 0.21 | 0.11-0.31 | -0.01 | -0.13-0.10 | 0.004* | 0.280 |  |

Abbreviations: LPS, lipopolysaccharide; MDD, Major Depressive Disorder; QIDS, Quick Inventory of Depressive Symptomatology; BMI, body mass index; SSRI, selective serotonin re-uptake inhibitor; IFN, interferon; IL, interleukin; MCP, monocyte chemotactic protein; MIP, macrophage inflammatory protein; MMP, matrix metalloproteinase; TNF, tumor necrosis factor. ^a^ Based on analyses of covariance. ^b^ Ln-transformed factors presented back-transformed. ^c^ Severity: adjusted for basic and lifestyle and health factors (i.e. site, age, sex, smoking status, alcohol intake, physical activity and number of chronic diseases under treatment) plus depression severity (baseline QIDS score without overlapping specifier items). ^d^ BMI: adjusted for basic and lifestyle and health factors plus BMI. ^e^ SSRI use: adjusted for basic and lifestyle and health factors plus SSRI use. * Results survive the Benjamini-Yekutieli correction for multiple comparisons threshold of <0.012.

**Table S3.** Adjusted associations between basal and LPS-stimulated inflammation index with various dimensional anxiety constructs in patients with current (past 6 months) MDD, additionally adjusted for QIDS score, BMI and SSRI use.

|  | **Basal inflammation index** | | | **LPS-stimulated inflammation index** | | |
| --- | --- | --- | --- | --- | --- | --- |
|  | **β** | ***P*^a^** | **R^2^** | **β** | ***P*^a^** | **R^2^** |
| Anxious distress specifier score |  |  |  |  |  |  |
| QIDS score adjusted^b^ | -0.04 | 0.265 | 0.068 | 0.13 | 0.030 | 0.136 |
| BMI adjusted^c^ | 0.02 | 0.557 | 0.178 | 0.14 | 0.002* | 0.144 |
| SSRI use adjusted^d^ | 0.04 | 0.195 | 0.055 | 0.14 | 0.002* | 0.140 |
| Number of anxiety disorders |  |  |  |  |  |  |
| QIDS score adjusted^b^ | -0.01 | 0.860 | 0.068 | 0.08 | 0.080 | 0.132 |
| BMI adjusted^c^ | 0.02 | 0.599 | 0.176 | 0.10 | 0.023 | 0.132 |
| SSRI use adjusted^d^ | 0.03 | 0.358 | 0.055 | 0.09 | 0.038 | 0.130 |
| IDS anxiety arousal subscale |  |  |  |  |  |  |
| QIDS score adjusted^b^ | -0.01 | 0.750 | 0.068 | 0.05 | 0.469 | 0.127 |
| BMI adjusted^c^ | 0.03 | 0.311 | 0.178 | 0.09 | 0.046 | 0.130 |
| SSRI use adjusted^d^ | 0.08 | 0.021 | 0.059 | 0.10 | 0.044 | 0.129 |
| Beck Anxiety Inventory |  |  |  |  |  |  |
| QIDS score adjusted^b^ | 0.03 | 0.501 | 0.068 | 0.17 | 0.001* | 0.147 |
| BMI adjusted^c^ | 0.04 | 0.168 | 0.178 | 0.18 | <0.001* | 0.152 |
| SSRI use adjusted^d^ | 0.08 | 0.010* | 0.060 | 0.18 | <0.001* | 0.151 |
| Fear Questionnaire |  |  |  |  |  |  |
| QIDS score adjusted^b^ | 0.02 | 0.538 | 0.068 | 0.05 | 0.295 | 0.128 |
| BMI adjusted^c^ | 0.05 | 0.072 | 0.179 | 0.09 | 0.069 | 0.129 |
| SSRI use adjusted^d^ | 0.06 | 0.050 | 0.058 | 0.08 | 0.094 | 0.127 |
| MASQ anxious arousal scale |  |  |  |  |  |  |
| QIDS score adjusted^b^ | -0.02 | 0.639 | 0.068 | 0.11 | 0.053 | 0.161 |
| BMI adjusted^c^ | 0.00 | 0.951 | 0.177 | 0.13 | 0.011* | 0.161 |
| SSRI use adjusted^d^ | 0.04 | 0.272 | 0.055 | 0.12 | 0.017 | 0.168 |
| Anxiety Sensitivity Index |  |  |  |  |  |  |
| QIDS score adjusted^b^ | -0.04 | 0.300 | 0.069 | 0.07 | 0.200 | 0.156 |
| BMI adjusted^c^ | -0.01 | 0.798 | 0.177 | 0.09 | 0.058 | 0.154 |
| SSRI use adjusted^d^ | 0.01 | 0.879 | 0.54 | 0.09 | 0.071 | 0.162 |
| Penn State Worry Questionnaire |  |  |  |  |  |  |
| QIDS score adjusted^b^ | -0.05 | 0.171 | 0.070 | 0.05 | 0.356 | 0.153 |
| BMI adjusted^c^ | 0.01 | 0.761 | 0.177 | 0.09 | 0.069 | 0.152 |
| SSRI use adjusted^d^ | 0.01 | 0.893 | 0.054 | 0.08 | 0.094 | 0.160 |

Abbreviations: LPS, lipopolysaccharide; MDD, Major Depressive Disorder; QIDS, Quick Inventory of Depressive Symptomatology; BMI, body mass index; SSRI, selective serotonin re-uptake inhibitor; IDS, Inventory of Depressive Symptomatology; MASQ, Mood and Anxiety Symptoms Questionnaire. ^a^ Based on linear regression analyses. ^b^ Severity: adjusted for basic and lifestyle and health factors (i.e. site, age, sex, smoking status, alcohol intake, physical activity and number of chronic diseases under treatment) plus depression severity (baseline QIDS score without overlapping specifier items). ^c^ BMI: adjusted for basic and lifestyle and health factors plus BMI. ^d^ SSRI use: adjusted for basic and lifestyle and health factors plus SSRI use. * Results survive the Benjamini-Yekutieli correction for multiple comparisons threshold of <0.012.

**Table S4.** Pearson r correlations between various dimensional anxiety indicators in patients with current (past 6 months) MDD.

|  | **Anxious distress specifier score** | **Number of anxiety disorders** | **IDS anxiety arousal subscale** | **Beck Anxiety Inventory** | **Fear Questionnaire** | **MASQ anxious arousal subscale** | **Anxiety Sensitivity Index** | **Penn State Worry Questionnaire** |
| --- | --- | --- | --- | --- | --- | --- | --- | --- |
|  | **r** | **r** | **r** | **r** | **r** | **r** | **r** | **r** |
| Anxious distress specifier score |  |  |  |  |  |  |  |  |
| Number of anxiety disorders | 0.41 |  |  |  |  |  |  |  |
| IDS anxiety arousal subscale | 0.70 | 0.40 |  |  |  |  |  |  |
| Beck Anxiety Inventory | 0.75 | 0.45 | 0.74 |  |  |  |  |  |
| Fear Questionnaire | 0.48 | 0.48 | 0.46 | 0.52 |  |  |  |  |
| MASQ anxious arousal scale | 0.54 | 0.38 | 0.63 | 0.73 | 0.41 |  |  |  |
| Anxiety Sensitivity Index | 0.49 | 0.40 | 0.42 | 0.55 | 0.45 | 0.50 |  |  |
| Penn State Worry Questionnaire | 0.49 | 0.34 | 0.36 | 0.39 | 0.32 | 0.35 | 0.43 |  |

Abbreviations: MDD, Major Depressive Disorder; IDS, Inventory of Depressive Symptomatology; MASQ, Mood and Anxiety Symptoms Questionnaire. All Pearson r correlations have *P*<0.001.

**Table S5.** Pearson r correlations between basal and LPS-stimulated inflammatory markers in patients with current (past 6 months) MDD.

|  | CRP | IL-6 | TNF-α | Index | IFN-ɣ | IL-2 | IL-4 | IL-6 | IL-8 | IL-10 | IL-18 | MCP-1 | MIP-1α | MIP-1β | MMP-2 | TNF-α | TNF-β | Index |
| --- | --- | --- | --- | --- | --- | --- | --- | --- | --- | --- | --- | --- | --- | --- | --- | --- | --- | --- |
|  | **r** | **r** | **r** | **r** | **r** | **r** | **r** | **r** | **r** | **r** | **r** | **r** | **r** | **r** | **r** | **r** | **r** | **r** |
| **Basal inflammatory markers** | | | | |  |  |  |  |  |  |  |  |  |  |  |  |  |  |
| CRP |  |  |  |  |  |  |  |  |  |  |  |  |  |  |  |  |  |  |
| IL-6 | 0.25^**^ |  |  |  |  |  |  |  |  |  |  |  |  |  |  |  |  |  |
| TNF-α | 0.14^**^ | 0.11^**^ |  |  |  |  |  |  |  |  |  |  |  |  |  |  |  |  |
| Index | 0.70^**^ | 0.67^**^ | 0.64^**^ |  |  |  |  |  |  |  |  |  |  |  |  |  |  |  |
| **LPS-stimulated inflammatory markers** | | | | |  |  |  |  |  |  |  |  |  |  |  |  |  |  |
| IFN-ɣ | -0.03 | -0.08 | 0.092 | -0.02 |  |  |  |  |  |  |  |  |  |  |  |  |  |  |
| IL-2 | 0.09 | 0.03 | 0.07 | 0.08 | 0.46^**^ |  |  |  |  |  |  |  |  |  |  |  |  |  |
| IL-4 | 0.02 | 0.12^**^ | -0.00 | 0.06 | 0.16^**^ | 0.30^**^ |  |  |  |  |  |  |  |  |  |  |  |  |
| IL-6 | 0.11^*^ | -0.07 | 0.14^**^ | 0.08 | 0.68^**^ | 0.55^**^ | 0.14^**^ |  |  |  |  |  |  |  |  |  |  |  |
| IL-8 | 0.19^**^ | 0.16^**^ | 0.10^*^ | 0.21^**^ | 0.24^**^ | 0.37^**^ | 0.26^**^ | 0.49^**^ |  |  |  |  |  |  |  |  |  |  |
| IL-10 | 0.03 | -0.06 | 0.01 | -0.02 | 0.28 | 0.11 | -0.07 | 0.36 | 0.12 |  |  |  |  |  |  |  |  |  |
| IL-18 | 0.22 | 0.13 | 0.22 | 0.26 | 0.33 | 0.37 | 0.20 | 0.39 | 0.44 | 0.15 |  |  |  |  |  |  |  |  |
| MCP-1 | 0.22^**^ | 0.17^**^ | 0.15^**^ | 0.25^**^ | 0.43^**^ | 0.30^**^ | 0.10^*^ | 0.56^**^ | 0.57^**^ | 0.39^**^ | 0.44^**^ |  |  |  |  |  |  |  |
| MIP-1α | 0.07 | 0.07 | 0.16^**^ | 0.14^**^ | 0.56^**^ | 0.42^**^ | 0.18^**^ | 0.76^**^ | 0.60^**^ | 0.31^**^ | 0.41^**^ | 0.62^**^ |  |  |  |  |  |  |
| MIP-1β | 0.09 | 0.06 | 0.17^**^ | 0.15^**^ | 0.56^**^ | 0.36^**^ | 0.07 | 0.74^**^ | 0.47^**^ | 0.50^**^ | 0.36^**^ | 0.65^**^ | 0.90^**^ |  |  |  |  |  |
| MMP-2 | 0.15^**^ | 0.12^*^ | 0.10^**^ | 0.16^**^ | 0.58^**^ | 0.61^**^ | 0.29^**^ | 0.70^**^ | 0.51^**^ | 0.36^**^ | 0.50^**^ | 0.69^**^ | 0.69^**^ | 0.68^**^ |  |  |  |  |
| TNF- α | 0.01 | -0.11^*^ | 0.16^**^ | 0.04 | 0.72^**^ | 0.44^**^ | 0.15^**^ | 0.79^**^ | 0.33^**^ | 0.33^**^ | 0.36^**^ | 0.40^**^ | 0.70^**^ | 0.68^**^ | 0.59^**^ |  |  |  |
| TNF- β | 0.11^*^ | -0.05 | 0.09 | 0.05 | 0.55^**^ | 0.66^**^ | 0.25^**^ | 0.68^**^ | 0.37^**^ | 0.36^**^ | 0.45^**^ | 0.45^**^ | 0.58^**^ | 0.56^**^ | 0.76^**^ | 0.61^**^ |  |  |
| Index | 0.14^**^ | 0.06 | 0.17^**^ | 0.16^**^ | 0.72^**^ | 0.66^**^ | 0.34^**^ | 0.86^**^ | 0.63^**^ | 0.46^**^ | 0.59^**^ | 0.72^**^ | 0.85^**^ | 0.82^**^ | 0.88^**^ | 0.77^**^ | 0.80^**^ |  |

Abbreviations: LPS, lipopolysaccharide; MDD, Major Depressive Disorder; CRP, C-reactive protein; IL, interleukin; TNF, tumor necrosis factor; IFN, interferon; MCP, monocyte chemotactic protein; MIP, macrophage inflammatory protein; MMP, matrix metalloproteinase. ** *P*<0.01. * *P*<0.05
