# Supplementary figures and images for: The role of anxious distress in immune dysregulation in patients with major depressive disorder
Source: Transl Psychiatry. 2017 Dec 8;7:1268. doi: 10.1038/s41398-017-0016-3 (PMC5802575; doi:10.1038/s41398-017-0016-3)

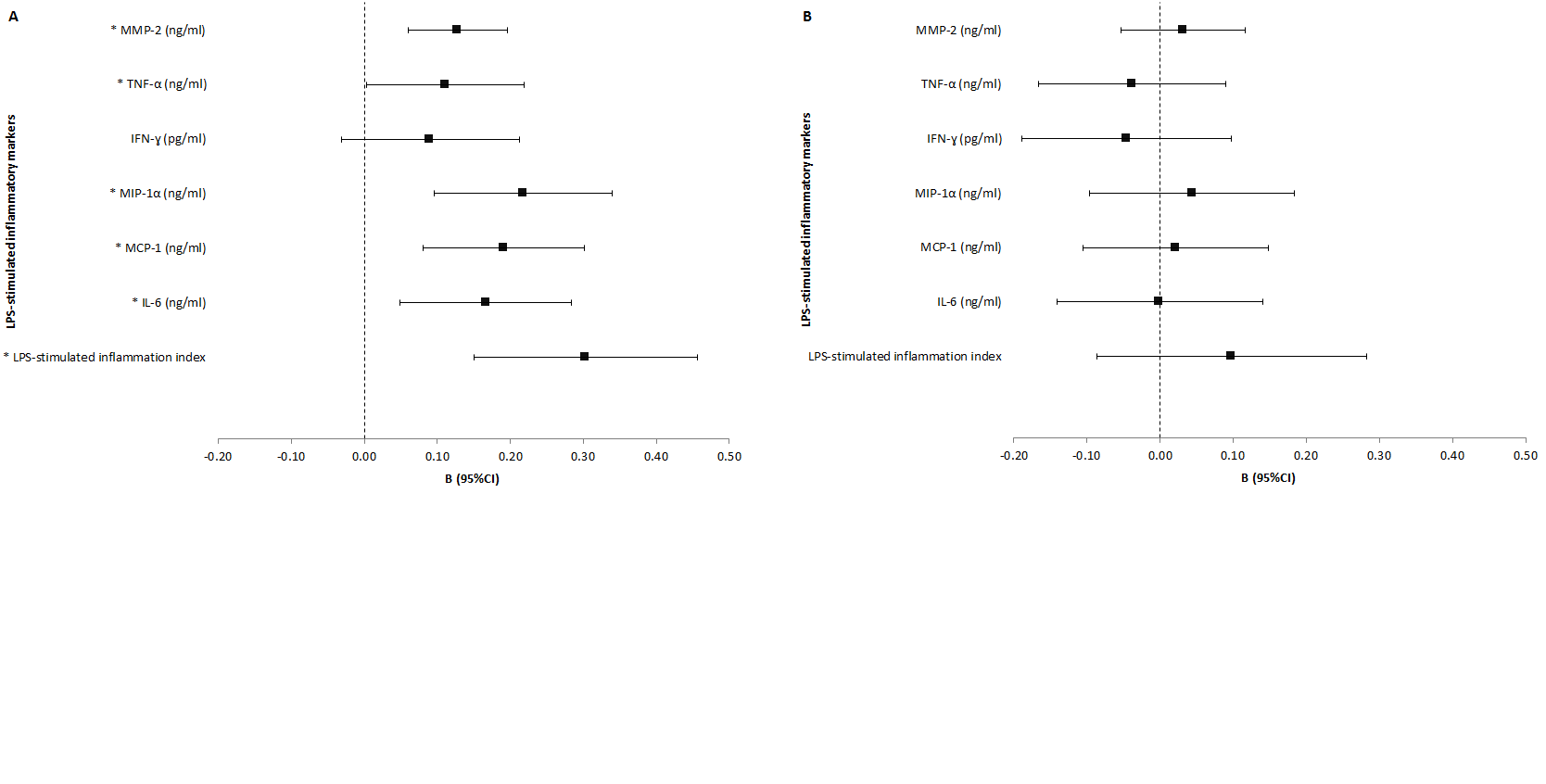

Supplement: Supplementary file 2 — Supplemental Figure S1 [file 41398_2017_16_MOESM2_ESM.tif]
